# NUCB2/Nesfatin-1 in the Blood and Follicular Fluid in Patients with Polycystic Ovary Syndrome and Poor Ovarian Response

**Published:** 2019

**Authors:** Zekiye Catak, Seyda Yavuzkir, Esra Kocdemir, Kader Ugur, Meltem Yardim, İbrahim Sahin, Esra Piril Agirbas, Suleyman Aydin

**Affiliations:** 1- Department of Clinical Biochemistry, University of Health Sciences, Elazig Fethi Sekin City Hospital, Elazig, Turkey; 2- Department of Obstetrics and Gynecology, School of Medicine, Firat University, Elazig, Turkey; 3- Department of Clinical Biochemistry, Kovancilar State Hospital, Elazig, Turkey; 4- Department of Internal Medicine (Endocrinology and Metabolism Diseases), School of Medicine, Firat University, Elazig, Turkey; 5- Department of Medical Biochemistry and Clinical Biochemistry (Firat Hormones Research Group), Medical School, Firat University, Elazig, Turkey; 6- Department of Medical Biology, Medical School, Erzincan Binali Yildirim University, Erzincan, Turkey; 7- School of Medicine, Medical School Student, Firat University, Elazig, Turkey

**Keywords:** PCOS, Infertility, Nesfatin-1, Follicular fluid, Glucose metabolism, Pregnancy

## Abstract

**Background:**

Failure to respond adequately to standard protocols and to recruit adequate follicles is called ‘poor ovarian response’. The relationships between metabolic alterations and NUCB2/Nesfatin-1 levels were explored in patients with polycystic ovary syndrome (PCOS) undergoing *in vitro* fertilization/intracytoplasmic sperm injection.

**Methods:**

This case-control study involved 20 infertile women with PCOS and 20 control women diagnosed as poor ovarian responders stimulated with a GnRH antagonist. Blood samples were taken during ovum pick-up and follicular fluids (FF) were obtained from a dominant follicle from the subjects. Samples were analyzed by using ELISA. Statistical analysis was performed with SPSS version 20. Data are expressed as means ± standard deviation (SD).

**Results:**

Blood NUCB2/Nesfatin-1 levels in PCOS were significantly lower (p= 0.011) while the NUCB2/Nesfatin-1 levels of FF in poor ovarian response (POR) were higher, but not statistically significant. Insulin, total testosterone, fasting glucose, homeostasis model assessment, and insulin resistance index in women with POR decreased when compared with PCOS. Blood NUCB2/Nesfatin-1 levels were significantly higher than FF NUCB2/Nesfatin-1 levels in both groups (p<0.001). Moreover, a positive correlation was detected between blood NUCB2/Nesfatin-1 and testosterone (p=0.602, r=0.304), HOMA-IR (p=0.252, r=0.384), BMI (p=0.880, r= 0.44) in PCOS, but it was not significant.

**Conclusion:**

NUCB2/Nesfatin-1 levels might be important in follicular growth in PCOS subjects undergoing IVF/ICSI with an antagonist protocol and NUCB2/Nesfatin-1 level could reliably help to predict poor ovarian response.

## Introduction

Polycystic Ovary Syndrome (PCOS) is a metabolic disease characterized by hyperandrogenism, polycystic ovarian morphology, ovulatory dysfunction (1, 2). It seems that 5–15% of women worldwide are affected by PCOS, which is the most widespread gynecological illness in women in their fertile period (3, 4). Despite many studies on the relevant genes that have a role in the modulation of gonadotropic, neuroendocrine and metabolic mechanisms for development of PCOS, the mechanisms underlying the development of the PCOS are not exactly known (4). Insulin resistance (IR) and increased insulin levels are also common in women with PCOS. There appears to be a correlation between insulin levels and severity of PCOS (5). NUCB2/Nesfatin-1 is a potent anorexigenic peptide that is produced in the hypothalamus, other brain regions, stomach and pancreas (1). NUCB2/Nesfatin-1 has antihyperglycemic effects (6). Blood NUCB2/Nesfatin-1 levels in type 2 diabetes mellitus (DM) and gestational DM patients also seem to be lower (1). This finding - the effects of NUCB2/Nesfatin-1 on energy balance, obesity, glucose metabolism and this being related to delayed puberty of lower NUCB2/Nesfatin-1 - showed that NUCB2/Nesfatin-1 in normal and pathological conditions may be important in the functioning of the ovaries and in reproduction (1, 3, 7, 8). Chung et al. have suggested that expression of NUCB2/Nesfatin-1 in pituitary may be regulated by ovarian estrogen and progesterone (8). It has also been suggested that T-helper17 cells are regulated by NUCB2/Nesfatin-1, and these cells are important in the uterus for in embryo implantation and the maintenance of pregnancy (8).

The follicular fluid that aids the development and maturation of oocyte provides a microenvironment composed of several regulatory proteins, hormones and metabolites (9). Effects of FF inclusion on oocyte maturation have led to the idea that NUCB2/Nesfatin-1 levels may be associated with ovarian dysfunction, such as oligo/anovulation, polycystic ovaries in PCOS patients. Today, many studies have been about whether NUCB2/Nesfatin-1 has a role in the development of PCOS. An increased NUCB2/Nesfatin-1 level related to development of PCOS has been intimated by Ademoglu et al. (10). In contrast, lower levels of NUCB2/Nesfatin-1 have been measured by others that may contribute to the development of PCOS (1, 3). These differing results may be due to genetic variation, patient selection, study design and/or experimental conditions (6). But there have been no reports as to whether the presence of NUCB2/Nesfatin-1 in FF and NUCB2/Nesfatin-1 in blood is related to the pathophysiology of PCOS or ovarian dysfunction in PCOS patients. Furthermore, there have also been contradictory results regarding blood levels of NUCB2/Nesfatin-1 in PCOS (1, 10). Therefore, our aim was to evaluate the difference of blood and FF NUCB2/Nesfatin-1 levels in PCOS patients and poor ovarian response (POR).

## Methods

This retrospective case-control study was conducted with 40 patients admitted at the IVF center, Firat University Hospital, Turkey. Twenty participants were diagnosed with PCOS according to the Rotterdam criteria (Who completed 2 of them; oligo/anovulation, clinical/biochemical hyperandrogenism, and polycystic ovaries). Diagnosis of polycystic ovaries was based on ultrasound criteria of polycystic ovaries that had 12 or more follicles in each ovary measuring 2–9 *mm* in diameter, and/or >10 *ml* ovarian volume. The other 20 participants were defined as poor responders based on the ESHRE criteria. Poor responders were identified as patients who produce a lesser number of oocytes than 25% of their respective age group. That is POR met the last 2 of 3 ESHRE criteria, high maternal age (≥40 years) or any of other risk factors for POR, a prior POR (≤3 oocytes after stimulation) and an unusual ovarian reserve test (i.e. AFC, 5–7 follicles or AMH, 0.5–1.1 *ng/ml*). None of the participants were using antihyperlipidemic, anti-diabetic, anti-androgenic, anti-hypertensive or glucocorticoids drugs that could affect NUCB2/Nesfatin-1 levels. Women with PCOS with higher number of oocytes than 25% of their respective age group were considered as the control group. Informed consent was obtained from all individual participants included in the study. This study was approved by the ethics committee of Firat University (Date: 06.12.2018; Issue number: 16).

### Collection of samples:

Patients undergoing assisted reproductive technology (ART) by standard ovarian stimulation protocols were recruited to collect follicular fluid (FF). In other words, when oocytes were taken from all the women undergoing ovarian stimulation for IVF/ICSI, FF was pooled. FF was obtained from mature sized follicles (≥16 *mm* diameter). Details regarding the stimulation parameters, oocyte retrieval and FF preparation and storage have been previously described (11). At the same time, venous blood samples were collected for NUCB2/Nesfatin-1 measurement as previously described (12). Biologic samples were centrifuged for 5 *min* at 4,000 *rpm* (1792 *g*). The obtained samples were stored at – 80*°C* until analysis by placing them into Eppendorf tubes. Blood glucose, insulin, estradiol, testosterone, LH and FSH levels were analyzed in the biochemistry laboratory of Firat University Hospital. Blood samples were taken from poor responders on day 3 of their menstrual period, and from women with PCOS on the 2nd–5th day of the progesterone withdrawal bleeding.

### ELISA analysis:

NUCB2/Nesfatin-1 in blood samples and FF were analyzed with a Human NUCB2/Nesfatin-1 ELISA kit (Elabscience Biotechnology, China) by ChroMate, Microplate Reader P4300 (Awareness Technology, USA). The coefficient of variance (CV) values were given as <10 by the kit manufacturers. The measurement interval of the kit was 15.6–1000 *pg/ml* NUCB2/Nesfatin-1, converted to *ng/ml* based on most research in this field for NUCB2/nesfatin-1. Therefore, our results could be compared more readily with previous reports that investigated NUCB2/nesfatin-1 in numerous diseases. Most kits are produced to be blood-specific; therefore, for the first time, an attempt was made to evaluate whether NUCB2/nesfatin-1 kits (Elabscience Biotechnology, China) could measure NUCB2/nesfatin-1 in human FFs at the same sensitivity as the blood level. Assay validation has been done according to previously published work by Aydin (12). The kit (Catalog no: E-EL-H2373) measured NUCB2/Nesfatin-1 in FF at the same sensitivity as the blood NUCB2/nesfatin-1 level.

### Statistical analysis:

All parameters were analyzed by the SPSS (SPSS Inc., Chicago, IL, USA) soft ware, version 20. The variables were investigated using visual (Histograms/probability plots) and analytical (Kolmogorov-Smirnov/Shapiro-Wilk’s) tests as to whether or not they are normally distributed. Since NUCB2/Nesfatin-1 measurements were not normally distributed, the Mann-Whitney U test was used to compare NUCB2/Nesfatin-1 levels between the groups. Correlation analyses used the Spearmen test. A power analysis was also used to estimate the minimum sample size required for this case-control study. For the multivariate analysis, the possible factors identified with univariate analyses were further entered into the logistic regression analysis to determine independent predictors of patient outcomes. Hosmer-Lemeshow goodness of fit statistics was used to asses model fit. Data are presented as mean± standard deviation (SD). Differences between groups were considered significant when p<0.05.

## Results

The demographic and biochemical characteristics of patients are given in table 1. Patients with PCOS were detected as younger (Nearly 4 years) in terms of age difference between the groups (p<0.001). Insulin, total testosterone, fasting glucose and HOMA-IR levels were significantly lower in women with POR than PCOS. There was no significant difference in estradiol, FSH and LH levels between the groups. Although BMI was higher in the PCOS group, this difference was not significant between the groups. A positive correlation between blood NUCB2/Nesfatin-1 and testosterone (p=0.602, r=0.304), HOMA-IR (p=0.252, r=0.384), BMI (p=0.880, r=0.44) in PCOS was found, but it was not significant (Table 2). This showed that blood NUCB2/Nesfatin-1 levels were significantly higher than FF NUCB2/Nesfatin-1 levels in both groups (p<0.001). Blood NUCB2/Nesfatin-1 levels were lower in PCOS patients compared to the POR group, and was statistically significant (p=0.011; table 3).

**Table 1.** Demographic, biochemical, and clinical characteristics of PCOS and POR

	**PCOS (n:20)**	**POR (n:20)**	***p* value**
**Age**	30.96±2.93	35.6±2.88	<0.001^*^
**Body mass index (*kg/m^2^*)**	26.92±4.92	25.43±4.6	0.337
**Infertility duration (*yr*)**	6.99±0.65	12.93±1.38	0.003^*^
**Day 3 FSH (*mU/ml*)**	5.70±1.1	9.74±4.83	0.052
**Day 3 LH (*mU/ml*)**	6.95±4.69	5.76±2.09	0.623
**Day 3 E2 (*pg/ml*)**	43.09±28.38	51.87±40.47	0.445
**Total testosterone (*ng/dl*)**	74.91±3.14	35.24±6.36	<0.001^*^
**HOMA-IR**	4.62±3.24	2.21±1.4	0.024
**Fasting insulin (*mU/ml*)**	19.14±1.48	12.38±1.29	<0.001^*^
**Fasting glucose (*mg/dl*)**	94.63±3.32	89.9±6.12	0. 012^*^
**Initial dose of rhFSH (*IU*)**	277.37±63.45	349.20±44.36	0.065
**Total dose of rhFSH (*IU*)**	2559±862.5	1986.1±960.4	<0.001^*^
**Duration of rhFSH (day)**	8.98±1.09	8.74±2.03	0.446
**E2 on the day of HCG (*pg/ml*)**	4188.8±1836.0	356.5±126.2	<0.001^*^
**Total oocyte number**	20.21±1.82	3.48±0.19	<0.001^*^
**2 PN**	10.99±6.43	2.07±0.79	<0.001^*^
**Fertilization rate (%)**	62.8	54.1	<0.001^*^
**Embryo transfer number**	1.95±0.28	1.61±0.47	0.005^*^
**Implantation rate (%)**	32.3	18.4	<0.001^*^
**Clinical pregnancy rate (%)**	58	28	<0.001^*^

rhFSH: recombinant human follicle-stimulating hormone; HCG: human chorionic gonadotropin; HOMA-IR: homeostasis model assessment for fasting insulin resistance; LH: Luteinizing hormone; PCOS: polycystic ovary syndrome.

**Table 2.** Correlation coefficients (*r*) between NUCB2/Nesfatin-1 levels and measured parameters in PCOS participants

**Independent variables**	**Blood NUCB2/Nesfatin-1**		**FF NUCB2/Nesfatin-1**	
	***r***	***p***	***r***	***p***
**BMI**	0.44	0.880	−0.212	0.440
**Day 3 LH**	−0.312	0.113	−0.232	0.483
**HOMA-IR**	0.384	0.252	0.119	0.564
**Testosterone**	0.314	0.602	−0.142	0.402
**Blood NUCB2/Nesfatin-1**	NA	NA	0.404	0.062

BMI: body mass index; FF: follicular fluid; HOMA-IR: homeostasis model assessment for fasting insulin resistance; LH: luteinizing hormone; NUCB2: nucleobindin 2; NA: not applicable.

**Table 3.** Blood and follicular fluid NUCB2/Nesfatin-1 of PCOS and POR

**NUCB2/Nesfatin-1**	**POR-B**	**PCOS-B**	***p***	**POR-FF**	**PCOS-FF**	***p***
**Median (*pg/ml*)**	2833.30	2291.70	0.011^*^	1632.80	1666.70	0.783
**Interquartile range**	500.01	666.70		572.90	572.93	

NUCB2: nucleobindin 2; PCOS-B: polycystic ovary syndrome – blood; PCOS-FF: blood polycystic ovary syndrome – follicular fluid; POR-B: poor ovarian response– blood; POR-FF: poor ovarian response – follicular fluid.

Results of correlation analyses showed that there were significant positive correlations between blood and FF NUCB2/Nesfatin-1 levels of POR, but not in women with PCOS (Table 4). Estimated relative risk is indicated by the odds ratio and the 95% confidence interval is also indicated in table 5.

**Table 4.** Correlation coefficients (r) between blood NUCB2/Nesfatin-1 levels and FF NUCB2/Nesfatin-1 in participants with PCOS and POR

	**PCOS-B**		**POR-B**	
	***r***	***p***	***r***	***p***
**(FF NUCB2/Nesfatin-1) – (NUCB2/Nesfatin-1)**	0.404	0.062	0.675	0.005^*^

FF: follicular fluid; NUCB2: nucleobindin 2; PCOS-B: polycystic ovary syndrome – blood; POR-B: poor ovarian response – blood.

* p<0.05 statically significant

**Table 5.** Estimated relative risk indicated by the odds ratio and the 95% confidence interval

**Risk factor**	RR^*^(95% confident interval)	p** value
**Age**	0.487(0.291–0.815)	0.006
**BMI**	0.907(0.723–1.137)	0.396
**Nesfatin-1**	1001(0.998–1.005)	0.475

* RR: The value represents the estimated relative risk indicated by the odds ratio and the 95% confidence interval

## Discussion

Even though some women emotionally or mentally were ready for pregnancy, they cannot be pregnant due to defective folliculogenesis. Defective folliculogenesis is very common in PCOS patients; its underlying cause is not fully understood. However, there is a link between PCOS and energy balance. The recently discovered hormone, NUCB2/Nesfatin-1, inhibits food intake and affects energy balance, insulin signaling and glucose metabolism (13). This hormone is ubiquitously produced in biological organs and tissues, including hypothalamus, pancreas, stomach, seminal gland, testis, uterus and ovary (14).

In line with this information, NUCB2/nesfatin-1 has been investigated in patients with PCOS undergoing *in vitro* fertilization/intracytoplasmic sperm injection (IVF/ICSI) with a gonadotropin-releasing hormone (GnRH) antagonist protocol. Blood NUCB2/nesfatin-1 levels were significantly higher in patients with POR than PCOS. Our data with respect to NUCB2/Nesfatin-1 levels are similar to previous studies in which blood NUCB2/Nesfatin-1 levels were evaluated in PCOS patients and in the other cases of insulin resistance like type 2 diabetes, gestational diabetes or morbid obesity (1, 3, 10, 15–17).

Although Deniz et al. suggested that higher BMI, higher glucose levels or hyperinsulinemia may be the result of reduced NUCB2/Nesfatin-1 in PCOS, it is unclear how NUCB2/Nesfatin-1 levels decrease in PCOS patients (1). NUCB2/Nesfatin-1 has an anti-hyperglicaemic effect due to its peripheral actions (18). Likewise, several studies indicate that NUCB2/Nesfatin-1 levels are lower in the patients with type 2 diabetes mellitus (DM), and gestational DM.

Lower levels of NUCB2/Nesfatin-1 lead to delayed of puberty in rats (7). In contrast, Gao et al. found that gonadotropin synthesis in the pituitary decreases after injection of NUCB2/Nesfatin-1 (19). Therefore, it was thought that NUCB2/Nesfatin-1, hypothalamic satiety peptide, could have a role in energy balance, glucose metabolism and gonadal functions and in development of PCOS (20). Moreover, disturbed secretion of some biochemical compounds and hormones in PCOS patients supports these findings (21).

An energy regulating hormone (E.g. leptin) in FF can be a predictor of successful IVF (22). Leptin levels of patients with PCOS who became pregnant had lower levels than other patients with PCOS who did not have a successful pregnancy (23, 24). In this sense, NUCB2/Nesfatin-1 had to be investigated in the FF. However, it was found that there was no significant difference in FF NUCB2/Nesfatin-1 levels between the groups. NUCB2 mRNA has been identified in human ovaries. Catak et al. demonstrated the synthesis of NUCB2/Nesfatin-1 in rat seminal gland, testis, uterus and ovary by ELISA and immunohistochemical methods (25).

The reason for the significant relationship between FF NUCB2/nesfatin-1 levels and PCOS remains currently unknown. The failed reproductive outcomes that may be seen in PCOS may be mediated by blood NUCB2/nesfatin-1 or other molecules, such as insulin and leptin, apart from FF NUCB2/nesfatin-1 levels, as mentioned above. The impact of insulin resistance and hyperinsulinemia on follicle development in women undergoing IVF/ICSI was reported by Fedorcsak et al. (26, 27). In line with their finding, by reducing the rates of pregnancy and fertilization in rats, obesity secondary to metabolic disorders can adversely affect the outcome of IVF/ICSI (28–30).

## Conclusion

In light of our findings, it is concluded that measurement of circulating NUCB2/nesfatin-1 might support the potential role of this peptide as a peripheral marker of follicular well-being. NUCB2/Nesfatin-1 might be also involved in our understanding of the unknown aspects of follicular development in patients with PCOS undergoing treatment with assisted reproductive technology. Undoubtedly, more studies with bigger sample size are needed, especially with regard to IVF/ICSI outcomes like the relationship between the quality of oocytes, embryos or pregnancy rate and follicular fluid nesfatin-1 levels.
